# Alternative Splicing of FOXP3—Virtue and Vice

**DOI:** 10.3389/fimmu.2018.00530

**Published:** 2018-03-13

**Authors:** Reiner K. W. Mailer

**Affiliations:** ^1^Institute of Clinical Chemistry and Laboratory Medicine, University Medical Center Hamburg-Eppendorf, Hamburg, Germany; ^2^Cardiovascular Medicine Unit, Department of Medicine, Karolinska Insititutet, Stockholm, Sweden

**Keywords:** FOXP3, isoform, alternative splicing, FOXP3Δ2, FOXP3Δ2Δ7, Treg cells, Th17 cells, antisense oligonucleotides

## Abstract

FOXP3 is the lineage-defining transcription factor of CD4+ CD25+ regulatory T cells. While many aspects of its regulation, interaction, and function are conserved among species, alternatively spliced FOXP3 isoforms are expressed only in human cells. This review summarizes current knowledge about alternative splicing of FOXP3 and the specific functions of FOXP3 isoforms in health and disease. Future perspectives in research and the therapeutic potential of manipulating alternative splicing of FOXP3 are discussed.

## Introduction

The immune system’s ability to tolerate structures recognized as self or non-pathogenic non-self is mediated by immunosuppressive mechanisms. Arguably the most effective suppressors of immune responses are CD4+ CD25+ regulatory T (Treg) cells (1–5). Phenotype and function of human Treg cells depend on the expression of their lineage-defining master transcription factor forkhead box P3 (FOXP3) (6). Ectopic expression of FOXP3 *via* retroviral transduction induces a Treg cell-like expression profile in human and, across species barriers, in mouse CD4+ CD25− T cells (7, 8). *Vice versa* the absence of functional FOXP3 is causative for severe autoimmune diseases and allergy in patients suffering from immunodysregulation, polyendocrinopathy, enteropathy, X-linked (IPEX) syndrome (9, 10). In mice, the homologous transcription factor Foxp3 (indicated as murine protein by the lower case letters; a consensus followed by most, but not all researchers) exerts the same function and truncated Foxp3 protein resulting from of a 2 bp insertion, that generates a frameshift and premature stop codon, leads to the *scurfy* phenotype, similar to IPEX syndrome (11). Detailed analysis over the last decades has been given a comprehensive view about mechanisms that regulate expression and protein functions of FOXP3/Foxp3 for Treg cell-mediated immunosuppression in health and disease (12). Here, a comparative overview is given at first, to apprehend the specific effects facilitated by human FOXP3 isoforms.

## FOXP3 Versus Foxp3

**Figure 1:**
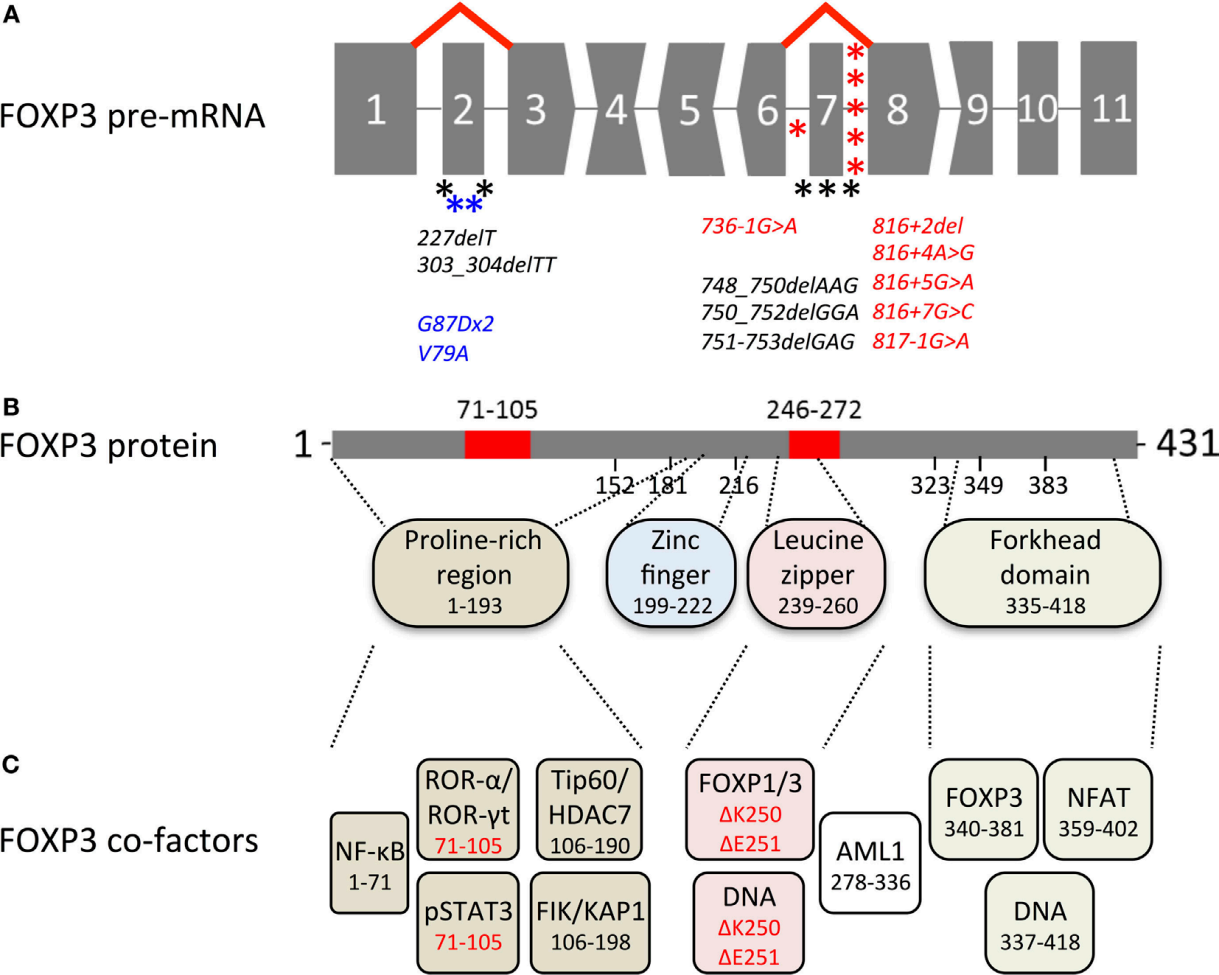
Overview of genetic, transcriptional, and functional features of human FOXP3. **(A)** Structure of the *FOXP3* pre-mRNA. Codons interrupted by introns are represented by convex and concave shapes for 1 and 2 nucleotides overhang, respectively. Alternative splicing of exon 2 and exon 7 is displayed by red lines; asterisks mark immunodysregulation, polyendocrinopathy, enteropathy, X-linked mutations that target alternatively spliced exons (black) or introns predicted to affect alternative splicing (red) and carcinoma mutations in alternatively spliced exons (blue). **(B)** FOXP3 protein with alternatively spliced exons (red), numbers indicate the region or the first amino acid of a new exon; dashed lines indicate regions of functional domains. **(C)** Cofactors binding FOXP3 functional domains, color-coded as in **(B)** numbers indicate the binding region, isoform-specific interactions highlighted in red.

*FOXP3* transcripts consist of a 5′-untranslated region (exon-1 in humans and exons-2b, -2a, and -1 in mice, in front of the start codon in exon 1) and 11 protein-encoding exons (Figure 1A). Proteins translated from murine and human transcripts share 86.5% amino acid sequence identity. In both species, distinct functional domains (N-terminal proline-rich region, zinc finger, leucine zipper, and forkhead domain) were identified (Figure 1B). FOXP3 binds to GTAAACA motifs *via* the winged-helix structure of the c-terminal forkhead domain and requires dimerization of the transcription factor by the leucine zipper, whereas the N-terminal 181 amino acids prevent DNA binding in an autoinhibitory fashion (13). Foxp3 regulates gene expression at several hundred DNA-binding sites identified throughout the genome (14–16). Despite the similarities to murine Foxp3, >50% of DNA regions bound by human FOXP3 are species-specific (17) and were not detected in studies analyzing mouse CD4+ CD25+ Treg cells (15) or FoxP3 overexpressing cell lines (16). FOXP3 allows transcriptional repression and transcriptional activation as part of a multi-protein complex whose composition likely diversifies the affinity to and the mode of interaction with DNA-binding sites (Figure 1C). Moreover, in cooperation with FOXP3-associated chromatin-modifying enzymes FOXP3 stabilizes epigenetically the Treg cell phenotype and function. Several excellent reviews summarize FoxP3-bound cofactors and the corresponding FoxP3 binding regions (18–20). However, it should be noted that identified cofactors may be species- and context-specific as posttranscriptional and posttranslational modifications can affect the complex formation (see below).

### Transcriptional Regulation of FOXP3

Due to the importance of FOXP3 for immunoregulation, intensive research efforts have been undertaken to elucidate potentially therapeutic mechanisms that induce FOXP3 transcription in CD4+ T cells (21). Species comparison identified several conserved non-coding sequences (CNS) that regulate *FOXP3* mRNA transcription through chromatin modifications (22). Initiation of FOXP3 expression is controlled by CNS3, which is located at intron 1 and facilitates indispensable c-Rel-mediated transcription in Treg precursor cells (23–25). Followed by this pioneer element, FOXP3 expression is further enhanced and stabilized by CNS2. This region is located distal to the promoter in intron-1 and controls the heritable maintenance of FOXP3 expression through various transcription factors, including FOXP3 itself. The accessibility to CNS2 is ensured through demethylation of its CpG islands, termed Treg cell-specific demethylated region (TSDR), that has been used to assess the Treg cell stability qualitatively and quantitatively (26, 27). In addition, CNS1, at the proximal site of intron-1, is associated with transforming growth factor-β1 (TGF-β1)-mediated FOXP3 induction. Thus, the transcriptional and epigenetic landscape of Treg cells appears to be a mosaic independently generated by T cell antigen receptor (TCR) stimulation (28, 29), FOXP3 expression (30), and TGFβ1-mediated effects (31). In line with this, comprehensive studies utilizing an array of conditional knockout mice revealed that constant Foxp3 expression (32) as well as continuous stimulation of the TCR and CD28 co-stimulation (33–35) is required for Treg cell function.

### Activation-Induced FOXP3 Expression

Importantly, anti-CD3/anti-CD28 stimulation of naïve human CD4+ T cells is sufficient to induce FOXP3 expression transiently but lacks strong immunosuppressive capacity (36–40), whereas in mice the differentiation of suppressive pTreg cells *in vivo* or iTreg cells *in vitro* depends on the presence of TGF-β1 during TCR ligation (41). In human naïve T cells, however, TGF-β1 treatment fails to stabilize FOXP3 expression and to induce phenotype and function comparable to Treg cells (42). Nevertheless, activation-induced FOXP3 expression in non-Treg cells has been shown to restrain proliferation and cytokine expression intrinsically (43), suggesting that activation-induced FOXP3 slows down T cell responses to enable further immunomodulatory mechanisms. This implies that FOXP3 expression among human CD4+ T cells is less sufficient to delineate the suppressive Treg cell population. These divergent FOXP3 functions in comparison to murine Foxp3 may be related with another important difference between mice and men, i.e., the generation of human FOXP3 isoforms through alternative splicing, as FOXP3 isoforms are variably expressed and differ in their ability to imprint the Treg cell phenotype.

## Alternative Splicing

Alternative splicing is an important mechanism to diversify protein functions and has been observed as a common concept in multiple T cell proteins to adapt to TCR stimulation (44). During the synthesis of pre-mRNA by RNA polymerase II in the nucleus, a multi-protein complex, termed the spliceosome, removes introns, and combines exons to generate mature mRNAs (45, 46). This posttranscriptional modification process relies on the identification of consensus sequences at the 5′- and 3′-splice site by ribonucleoproteins. This step is regulated by exonic or intronic enhancer/silencer auxiliary elements, which represent promising targets for antisense gene therapy with splice-shifting oligonucleotides (47). Exons not recognized by the spliceosome are not inserted into the mRNA and isoforms derived from this alternatively spliced transcript lack the encoded protein region. Following the splicing process, exon–exon junction sites on the mRNA remain bound to the exon junction complex until the first translating ribosome removes these protein assemblies. This facilitates the degradation of incorrectly spliced mRNAs, in which intron retention or exon skipping occurred erroneously, *via* nonsense-mediated mRNA decay. This quality control mechanism detects premature translation–termination codons generated by a frameshift (stop codons that are preceding exon junction complexes on the mRNA) and degrades the faulty mRNA.

### Alternative Splicing of FOXP3

Importantly, the structure of the *FOXP3* gene limits the amount of possible exon deletions (Figure 1A), as only exon 2 and exon 7 have blunt 5′- and 3′- ends, whereas codon-interrupting sequences would cause frameshifts in FOXP3 isoforms with exon deletion (Δ indicates deleted exon in the transcript/protein) Δ3, Δ4, Δ6, Δ8, and Δ9. Deletion of exon 1 would erase the transcription start site, whereas Δ5 and Δ10, albeit possible without frameshift, would omit DNA-interacting sites (zinc finger and forkhead domain, respectively). Interestingly, the gene architecture of murine FoxP3 (429 aa) is identical to that in humans, suggesting that alternative splicing of coding exons could also take place in mice. However, neither FoxP3 isoforms nor differential usage of non-coding alternative exons was found in lymphoid tissues or in phenotypically distinct CD4+ CD25low hepatic Treg cells (48, 49), whereas in rat (429 aa), isoforms with alternative N-terminal region (429 + additional 59 aa, GenBank accession: BAJ05811.1) or C-terminal region (322 + alternative 13 aa, GenBank accession: BAJ05812) have been reported. Taken together, splicing control of the transcription factor appears to be species-specific and no model organism is currently known that expresses analogs to human FOXP3 splice variants in CD4+ T cells: full-length FOXP3 (FOXP3fl) and exon 2 (FOXP3Δ2) as well as exon 2 and exon 7 (FOXP3Δ2Δ7) lacking isoforms (8, 50). The exact molecular mechanism of alternative splicing of *FOXP3* pre-mRNA has not been revealed yet, but the analysis of intronic sequences (intron 2 and intron 7) with splice factor binding prediction software points toward heterogeneous nuclear ribonucleoproteins that might act as intronic splicing silencers (51). However, since the skipping of these exons is not quantitatively correlated, it is likely that several different factors affect alternative splicing exon-specifically. Furthermore, the current concept for the change of isoform ratios envisages that alternatively spliced exons are more likely included into the nascent transcripts when RNA polymerase II elongation is slowed down through methylation of CpG islands and/or histone deacetylation (52, 53). Along this line, initial *FOXP3* transcription of “closed” chromatin induced by TCR stimulation and mediated by c-Rel/CNS3 would favor FOXP3fl expression. In contrast, *FOXP3* transcription of “open” chromatin in *bona fide* Treg cells mediated by TSDR/CNS2 would favor exon skipping. Further research will be necessary to answer these questions and to pinpoint factors that regulate alternative splicing of FOXP3.

## All Exons Combined

### FOXP3 Isoform Ratios in Disease

The longest *FOXP3* transcript translates into FOXP3fl and allocates 20–30% of the total FOXP3 expression (mRNA and protein) in human CD4+ CD25+ Treg cells (8, 54, 55). We have recently demonstrated that the inclusion of exon 2 among cellular FOXP3 isoforms expressed by CD4+ T cells correlates with TCR stimulation *in vitro* (56). This activation-induced FOXP3 isoform profile has been detected by co-stainings with two different anti-FOXP3 antibody clones that recognize exon 2 and a non-spliced region of FOXP3 to calculate a ratio of either fluorescence intensity per individual cell. This flow cytometric approach revealed a dose-dependent increase of the average (FOXP3 exon 2/FOXP3 total) ratio in CD4+ T cells treated with anti-CD3 antibodies, low-density lipoprotein or phorbolester. Besides these relative changes in both FOXP3low non-Treg cells as well as FOXP3high Treg cells, the total FOXP3+ T cell population is stained proportionally with both antibodies (56, 57), unless alternative splicing of exon 2 is artificially enforced by splice-shifting antisense oligonucleotides (ASO) (54). This strongly indicates that CD4+ T cells naturally express FOXP3 isoforms lacking exon 2 only in combination with FOXP3fl, but not alone. Along this line, gating analyzes to describe cell populations stained positively for FOXP3 total but negatively for FOXP3 exon 2 may be misleading and define FOXP3 isoform ratios improperly, as described for peripheral blood from patients with giant cell arteritis (58), vasculitis (59), or anti-phospholipid syndrome (60). Thus, instead of population analysis, it appears that isoform expression ratios calculated per cell represent a more liable and accurate parameter to investigate the relative expression of FOXP3 exon 2 by flow cytometry.

Moreover, the expression of FOXP3 isoforms has been assessed in immunoblots using an isoform-sensitive anti-FOXP3 antibody clone. Chen et al. have shown that FOXP3 expression in CD4+ CD25+ T cells negatively correlated with C–C chemokine ligand 3 serum concentrations derived from peripheral blood of psoriasis patients (61). Although not analyzed separately, it appears that the (upper FOXP3fl/lower FOXP3Δ2 band) ratio is decreasing in remission, whereas relatively more FOXP3fl is detected in patients with refractory lesions or chronic disease development. At this stage, it is unclear whether the altered FOXP3 isoform ratio in psoriasis depends on activated non-Treg cells or impaired Treg cells. However, in the same report FOXP3 expression has been shown to depend on the AKT pathway, which is activated downstream of the TCR and abrogates FOXP3-mediated suppression if constitutively activated (62). Strong activation reduced, whereas weak activation of the AKT pathway increased the [upper FOXP3fl/lower FOXP3Δ2 band] ratio in CD4+ CD25+ T cells *in vitro* (61). This corroborates our recent findings that TCR stimulation promotes an activation-induced FOXP3 isoform profile (56).

In addition, the association of impaired FOXP3 isoform ratios with disease has been investigated in several studies through quantification of *FOXP3* splice variants by real-time PCR. In rheumatoid arthritis patients, the increase of *FOXP3* transcripts including exon 2 exceeds that of *FOXP3* transcripts excluding exon 2 in isolated CD4+ T cells from blood (2.3- to 3.2-fold) and synovial fluid (3.6-fold) (63–65). Moreover, this shift is associated with increased expression of activation marker CD25, decreased expression of Treg cell marker CTLA-4 and can be reversed through decoy TNF-α receptor treatment. Likewise, inclusion of exon 2 in *FOXP3* mRNAs significantly increases in blood from coronary artery disease patients compared to healthy controls (56) and the same trend is seen in blood from patients with autoantibodies (66), rheumatoid arthritis (67), and biopsies from inflammatory bowel disease patients (57).

Taken together, these results suggest that the relative increase of FOXP3fl correlates with *de novo* induction of FOXP3 in activated CD4+ T cell and is associated with autoimmune and inflammatory diseases. Additional studies are required to investigate FOXP3 isoform patterns in resting, effector and memory subpopulations of Treg and non-Treg cells.

### FOXP3fl-Specific Functions

The presence of exon 2 in FOXP3fl enables isoform-specific functions. Interestingly, exon 2 harbors a nuclear export signal (NES), which promotes the relocation of FOXP3fl into the cytoplasm following its TCR stimulation-dependent expression in CD4+ CD25− T cells (68). In the same report, Magg et al., have shown that targeted mutation of FOXP3’s NES promotes Treg cell-associated gene transcription and suppressive capacity. Thus, nuclear localization of FOXP3fl supports the Treg cell phenotype, whereas cytoplasmic FOXP3fl facilitates additional isoform-specific functions, such as exon 2-specific associations with cofactors. Importantly, an interaction that has been demonstrated for human tumor-induced Treg cells is the association of signal transducers and activators of transcription 3 (STAT3) with the β-sheet region of exon 2 in FOXP3fl (69). In these cells, FOXP3fl binds to phosphorylated STAT3 to promote IL-10 transcription. Moreover, FOXP3fl recruits cytoplasmic histone acetyltransferase-1 into the nucleus to control epigenetic chromatin modifications at promoter sites. It is, therefore, tempting to speculate that this mechanism also contributes to the accessibility of CNS regions to imprint *FOXP3* transcription upon induction.

Another FOXP3fl-specific interaction is the association of exon 2 with Th17 cell lineage-defining transcription factors, i.e., retinoid acid receptor-related orphan receptors (ROR)-α and ROR-γt (70, 71). The LXXLL motif of FOXP3 exon 2 has been identified to interact with ROR-α and the expression of FOXP3fl is necessary to block ROR-γt-mediated Th17 cell differentiation in retroviral transduction experiments. Therefore, it has been proposed that human Th17 cells might predominantly express FOXP3Δ2. However, *ex vivo* analysis of Th17 cells from patients with Th17-related diseases reveals that IL-17 expression is rather associated with FOXP3fl (57, 72). This suggests that FOXP3 isoform-specific functions can be obscured upon overexpression *in vitro* (73), because retro- or lentiviral gene transfer into primary T cells results in multiple infections of target cells, random integration sites, a marked overexpression of transduced genes under the control of viral promoters and activation-induced expression of endogenous FOXP3. To avoid interference by these factors, we analyzed FOXP3 isoform-specific functions by splice-shifting ASOs. Notably, we have found that these synthetic polymers effectively inhibit the inclusion of alternatively spliced exons in endogenous *FOXP3* mRNAs and that lack of exon 2 does not promote Th17 cell differentiation (54). Moreover, *IL-17* mRNA does not correlate with *FOXP3* transcripts expressing or lacking exon 2 in biopsies obtained from patients with Crohn’s disease or peripheral blood from patients with coronary artery disease (54, 56). Thus, the FOXP3fl-ROR-interaction is dispensable for Th17 cell differentiation blockade and may rather abrogate the FOXP3fl-mediated control of cytokine expression (74).

So far, only few FOXP3fl-specific functions have been reported. As mentioned above, FOXP3fl increases the p-STAT3-dependent transcription of IL-10 (69). In addition, FOXP3fl induces the expression of PIM2, a serine/threonine kinase that is involved in the phosphorylation of murine Foxp3 and the expansion of human FOXP3fl-transduced T cells (75, 76). Both mechanisms await further investigation to clarify their impact on human Treg cell functions. In general, the Treg cell phenotype that is induced by FOXP3fl or FOXP3Δ2 expression in naïve CD4+ T cells appears to be overlapping, as NFAT/NF-κB-mediated transcriptional control, Treg cell marker expression as well as suppressive capacity is comparable in transduced human and murine T cells (8, 50, 55). However, the isoform-specific spatial and temporal expression of FOXP3fl indicates that the amount of FOXP3fl may influence the T cell fate *in vivo*.

## Exon 2 Skipping

Around 70% of *FOXP3* transcripts in human CD4+ CD25+ T cells are expressed as the alternative splice variant *FOXP3Δ2* (8, 54, 55). FOXP3Δ2 lacks the NES in exon 2 and is less prone for relocation into the cytoplasm compared to FOXP3fl (68). Despite the predominant expression of this isoform in Treg cells, relatively few studies have taken alternative splicing of human FOXP3 into account and investigated individual isoforms independently. Thus, specific features of FOXP3Δ2, such as DNA occupancy and cofactor binding to the exon1–exon3 interface, remain largely unknown. Moreover, the coexpression of FOXP3 isoforms and the possibility that a multiprotein complex may contain different FOXP3 isoforms could hamper the assignment of isoform-specific functions in Treg cells from healthy donors. Furthermore, the detection of FOXP3 isoforms relies on suitable isoform-sensitive anti-FOXP3 antibody clones that bind specific epitopes. Although alternative splicing can only occur at exon 2 and exon 7 (see above), De Rosa et al. have reported four different bands by immunoblot analysis of iTreg cells (77). In this paper, the interference with metabolic pathways regulates the amount of induced FOXP3; FOXP3 isoform ratios, however, remain constant in real-time PCR and FOXP3fl-specific immunoblots with respect to the total FOXP3 expression. In contrast, the quantification of unidentified bands obtained from immunoblots utilizing anti-FOXP3 antibody clone PCH101 have been interpreted to depict different FOXP3 isoform ratios that may have compromised the conclusion. Thus, the FOXP3 isoform ratio of iTregs cells in comparison to *ex vivo* isolated Treg cells or *in vitro* generated effector T cells awaits further investigation.

### IPEX Mutations Targeting FOXP3 Exon 2

However, evidence for the function of FOXP3 exons can be deduced from reported IPEX mutations that affect alternative splicing or the protein sequence of exons (78). A cluster of IPEX mutations targeting the 5′ splice donor site of intron 1 (position 210 + 1/+2 nt) is predicted to cause aberrant splicing of exon 1 (47). However, two mutations are identified within exon 2 that lead to frameshifts and premature stop codons (227delT and 303_304delTT) (79–84). While the mutated *FOXP3fl* transcript is likely to be degraded by nonsense-mediated mRNA decay, alternative splicing of exon 2 should produce mRNA that encodes for functional FOXP3Δ2. Surprisingly, no Treg cells expressing FOXP3Δ2 alone have been detected in these IPEX patients by flow cytometry using isoform-sensitive anti-FOXP3 antibodies (80, 82). Compared to IPEX patients of similar age with diminished FOXP3 expression due to a mutation in the forkhead domain, the mutation of exon 2 caused a strong increase of CD45RO expression in peripheral CD4+ T cells (80). Moreover, FOXP3 profiles of the Treg cell phenotype population (CD4+ CD25+ CD127−) derived from IPEX patients and IPEX patients’ mothers have been used to reveal functional differences between specific mutations (82). CD4+ CD25+ CD127− T cells, that are FOXP3+ in healthy controls, lack FOXP3 expression in patients with a point mutation in the forkhead domain. The IPEX patient’s mother, who carries the wild type allel and the IPEX mutation on her respective X chromosomes, has about 50% FOXP3− T cells within the Treg cell phenotype population, suggesting that the IPEX mutation in T cells with inactivated wild type X-chromosome does not impair their development. In contrast, no FOXP3− T cells are found in the CD4+ CD25+ CD127− population from the female carrier of the IPEX mutation in exon 2. This suggests that FOXP3Δ2 lacks the ability to promote its own transcription during development and that imprinting of the CD4+ CD25+ CD127− FOXP3+ Treg cell phenotype requires the presence of a functional non-spliced FOXP3 N-terminus.

## Exon 7 Skipping

Another alternative splice variant, *FOXP3Δ2Δ7*, allocates 1–3% of the total *FOXP3* mRNA in human CD4+ CD25+ Treg cells (8, 54). The alternatively spliced isoform FOXP3Δ2Δ7 was first described in peripheral blood and was thought to reduce T cell activation (85). Later characterization showed that in contrast to FOXP3fl- and FOXP3Δ2-transduced murine CD4+ T cells, FOXP3Δ2Δ7 expression does not induce the Treg cell phenotype and fail to suppress proliferation of responder cells (8). These results have also been confirmed for human T cells in lentiviral FOXP3Δ2Δ7 transduction experiments and found that specifically in T cells this isoform is strictly expressed in the nucleus through the deletion of both NES located in exon 2 and exon 7 (68). Moreover, transgenic expression of the artificial murine equivalent, FoxP3δ2δ7, does not provide the ability for immune regulation and FoxP3δ2δ7 knock-in mice develop a *scurfy*-like lymphoproliferative disease (86).

### IPEX Mutations Targeting FOXP3 Exon 7

The importance of exon 7 for the proper function of the transcription factor is highlighted by many IPEX mutations that target the coding sequence (37, 82, 87–92) as well as the flanking sequences intron 6 (92) and intron 7 (93–98), that control alternative splicing of exon 7 (Figure 1A). The mutations located in intronic sequences were predicted to facilitate aberrant splicing of exon 7 (78). In line with that, *FOXP3* mRNA lacking exon 7 has been detected in two patients with IPEX mutation 816 + 4A > G or 816 + 7G > C (95) and FOXP3 expression, albeit reduced, has been reported for IPEX mutations 816 + 7G > C and 817 − 1 G > A (95–97). Interestingly, malignant Treg cells from a Sézary syndrome patient expressed high amounts of FOXP3Δ2Δ7 and to a lesser extent FOXP3Δ2, but lacked FOXP3fl expression, indicating that FOXP3Δ2Δ7 fails to suppress the impaired activation and proliferation status (99).

FOXP3 exon 7 is part of the leucine zipper domain (Figure 1B) and the IPEX mutations ΔLys250 and ΔGlu251 have been shown to prevent FOXP3 homodimerization or FOXP1 heterodimerization (88, 100). However, the forkhead domain alone has been reported to form a stable domain-swapped dimer with DNA (101) and dimerization of FOXP3Δ2Δ7 with other isoforms has been intact in HEK293T cells (8). Of note, no FOXP3 expression has been detected in CD4+ T cells from FOXP3ΔGlu251 IPEX patients by flow cytometry using isoform-sensitive anti-FOXP3 antibodies (91), whereas patient-derived cell lines and transfected Jurkat or HEK293T cells readily express both mutants, human FOXP3ΔGlu251 or murine FoxP3ΔGlu250 (88, 102). These data highlight the cell type-specific expression, interaction and translocation of FOXP3, and indicate that in T cells impaired formation of homo-/heterodimers not only diminishes FOXP3 functions but also destabilizes its expression in total. In line with this notion, competing posttranslational modifications of the lysine residues in exon 7 have been found to support complex formation through acetylation or protein degradation through ubiquitination (103–105). The absence of exon 7 potentially abrogates this control mechanism and may lead to a swift change of the FOXP3 isoform ratio in conditions that are unfavorable for Treg cells. By this, the relatively low expression of FOXP3Δ2Δ7 may exert its dominant-negative effect to modulate Treg cell functions (8). The precise mechanism deployed to inhibit the immunosuppressive isoforms FOXP3fl and FOXP3Δ2 is unknown so far. FOXP3Δ2Δ7 has been shown to reduce NFAT- and NF-κB-mediated gene transcription as well as to interact with AML1 similar to the other FOXP3 isoforms (8). Based on this, it is plausible that FOXP3Δ2Δ7 neutralizes and sequestrates many cofactors while missing out a crucial component to facilitate immunosuppression, which could encompass the interaction with FOXP1 (88).

### Exon 7 Skipping in Th17 Cells

Interestingly, pro-inflammatory stimuli can decrease the function of human Treg cells and induce mixed phenotype profiles. In particular, Th17 cells share some developmental trails with Treg cells and sustain FOXP3 expression to inhibit Th1 cell differentiation (43). Inflammatory bowel disease is associated with the accumulation of FOXP3+ T cells and these cells have been found to preferentially express IL-17 or IL-17 in combination with TNF-α (57). Notably, Treg/Th17 cell plasticity is triggered by IL-1β/IL-2 signaling (106), conditions that also increase the expression of *FOXP3* transcripts lacking exon 7 (54). Moreover, deleting exon 2 and exon 7, but not exon 2 alone, from *FOXP3* pre-mRNA *via* splice-shifting ASOs promotes the differentiation of naïve T cells into Th17 cells (54). Thus, FOXP3Δ2Δ7 affects Th17 cell differentiation and may contribute to altered *FOXP3Δ2/FOXP3total* ratios observed in blood from Hashimoto’s thyroiditis patients or in intestinal biopsies from celiac disease patients (107, 108). Compared to healthy controls, *FOXP3* transcripts lacking exon 7 increase in synovial fluid of rheumatoid arthritis as well as in peripheral blood and biopsies from Crohn’s disease patients (54, 63). Furthermore, the expression of *IL17A* mRNA and *FOXP3* transcripts lacking exon 7 correlates in Crohn’s disease biopsies and peripheral blood from coronary artery disease patients (54, 56), indicating that skipping of exon 7 is a common event for IL-17 expression in diverse settings. Further studies will have to elucidate whether FOXP3 isoforms also play a role for IL-17-releasing non-CD4+ cells, e.g., FOXP3+ CD8+ T cells and recently described FOXP3+ eosinophils (109–111).

Taken together, alternative splicing of FOXP3 exon 7 abrogates the suppressive function of the transcription factor and promotes Th17 cell differentiation. The isoform FOXP3Δ2Δ7 may compete for cofactors and prevent complex formation; therefore, exon 7 represents a promising target to counteract other splice variants.

## FOXP3 Isoforms in Cancer

FOXP3 expression is not exclusively restricted to immune cells as mRNA and protein is also present in epithelial cells (112). Moreover, absence, mutation, or splice defects of FOXP3 have been reported in various types of carcinoma (113–116). FOXP3 represses the expression of different oncogenes (113–116), while overexpression of FOXP3, but not FOXP3ΔGlu251, decreases tumor cell proliferation (116, 117). Interestingly, some malignancies affected alternative splicing of FOXP3 (99, 113, 118–120). Of note, the numbering of FOXP3 exons differs between immunology and oncology literature. (The first exon is labeled exon-1 and exon 1 in immunological and oncological context, respectively.) Compared to FOXP3fl and FOXP3Δ2 expressing primary mammary epithelial cells, FOXP3fl is absent in breast cancer cell lines and increased exon skipping deleted exon 2, exon 7, or combinations of exon 2 and exon 3, or exon 2 and exon 7 (113). Moreover, the elevated expression of FOXP3Δ2 in bladder cancer causes chemotherapy resistance, facilitates development of more aggressive tumors and correlates inversely with overall survival (119). Furthermore, the *FOXP3* gene is directly hit by somatic mutations, some of which target exon 2 (G87Dx2 and V79A) (113, 120). Analysis of the V79A mutation, found in prostate cancer, revealed that mutated FOXP3fl fails to localize within the chromatin fraction, while wild-type FOXP3Δ2 lacks repression of MYC transcription and colony formation in spite of chromatin localization (120). Thus, biased expression of FOXP3Δ2 in epithelial cancers suggests that this isoform lacks the ability for cell cycle control and epigenetic regulation.

## Conclusion

**Figure 2:**
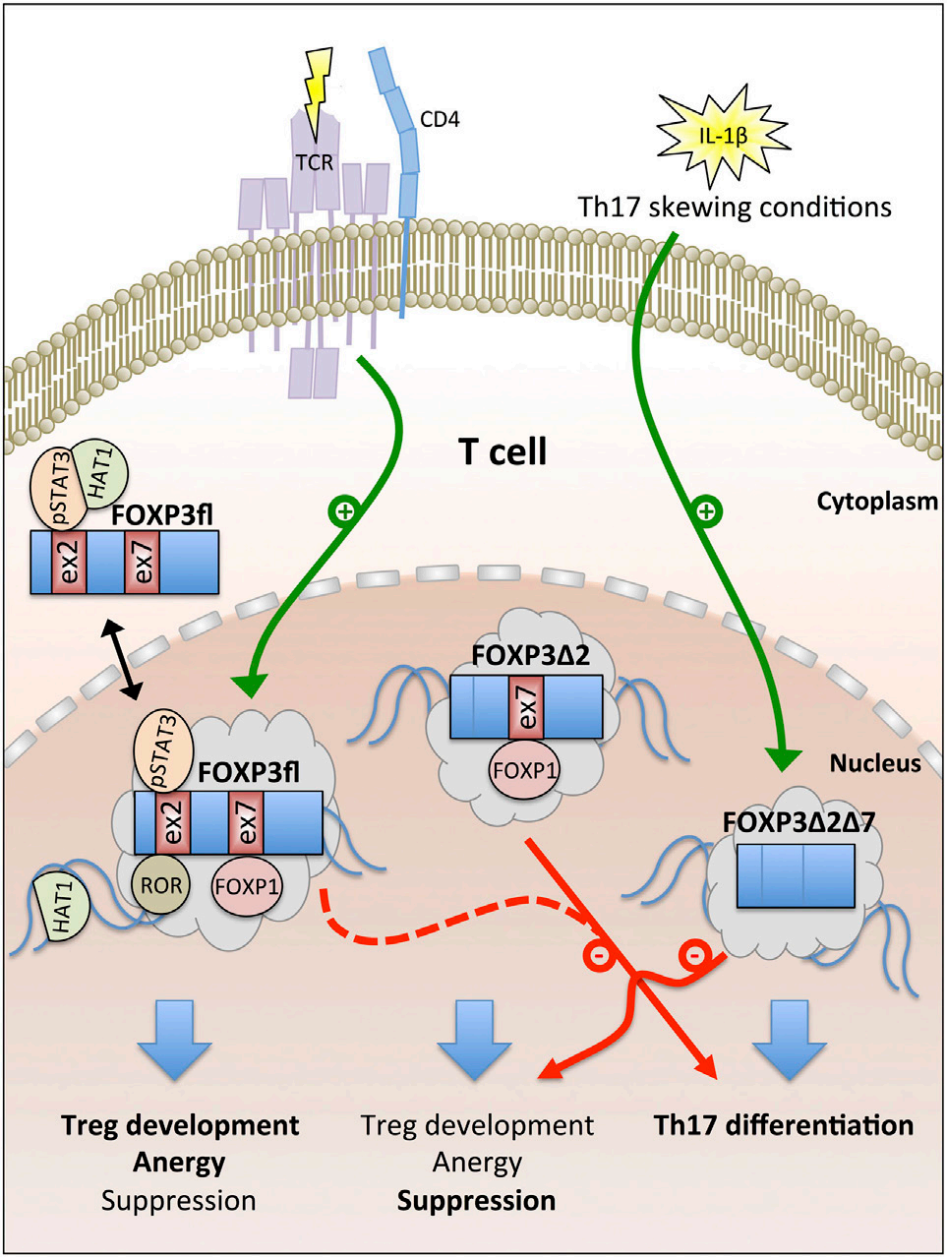
Overview of FOXP3 isoform functions. T cell antigen receptor (TCR) signaling promotes the generation of the full-length FOXP3 isoform FOXP3fl. Nuclear export signals (NES) in FOXP3fl enable recruitment of cytoplasmic cofactors [e.g., phosphorylated signal transducer and activator of transcription 3 (pSTAT3) and histone acetyltransferase-1 (HAT1)]. In the nucleus, exon 2 and exon 7 of FOXP3fl bind to retinoid acid receptor-related orphan receptors (ROR) and forkhead box P1 (FOXP1), respectively. FOXP3fl induces Treg cell development, T cell anergy, and suppression. FOXP3Δ2 lacks a NES in exon 2 and supports Treg cell-mediated suppression but fails to interact with pSTAT3 and ROR; both FOXP3fl and FOXP3Δ2 inhibit Th17 cell differentiation. IL-1β signaling induces the generation of FOXP3Δ2Δ7 that lacks NES in exon 2 and exon 7. FOXP3Δ2Δ7 promotes Th17 cell differentiation and acts as dominant-negative inhibitor of FOXP3fl and FOXP3Δ2.

In summary, cumulative evidence suggests that the expression of FOXP3fl is promoted by TCR signaling in CD4+ T cells and that higher FOXP3fl ratios in peripheral blood indicate inflammatory conditions. FOXP3fl is an intrinsic suppressor of proliferation in T cells and epithelial cells and exerts specific functions that are linked with epigenetic modifications imprinting the Treg cell lineage development. FOXP3Δ2 is more spread to the nuclear compartment than FOXP3fl, maintains primarily the suppressive phenotype in Treg cells and requires a pre-established Treg cell phenotype for stable expression. In contrast, nuclear FOXP3Δ2Δ7 correlates with IL-17 expression in peripheral blood and restricts the function of other FOXP3 isoforms *via* competition for cofactors (Figure 2).

## Future Perspectives

The generation of isoforms has a strong impact on functional properties of human FOXP3 and the analysis of the FOXP3 isoform profile helps to assess the cellular status. Thus, utilized anti-human FOXP3 antibody clones need to be specified to clarify whether FOXP3 isoform expression has been considered. The development of additional anti-FOXP3 antibodies specific for alternatively spliced regions and epitope mapping for existent antibody clones will improve future investigations of FOXP3-expressing cells, as there are currently no monoclonal anti-FOXP3 antibodies available that recognize exon 7 or Δ7 and the array of applications for Δ2-specific antibodies is limited (Table 1). However, real-time PCR techniques to quantify alternative splicing of exon 2 and exon 7 have provided insight into activation-induced and Th17-related FOXP3 isoform expression ratios in healthy and inflammatory conditions at cell population levels. Moreover, a novel approach that calculates the splicing ratio of FOXP3 exon 2 through co-stainings with antibodies recognizing exon 2 and a non-spliced region of FOXP3 by flow cytometry offers the possibility to observe differential alternative splicing on a cellular level. This method may help to elucidate FOXP3 isoform expression during thymic development and in peripheral T cell subpopulations in future studies.

**Table 1:** Epitopes of monoclonal anti-human FOXP3 antibody clones.

Clone	Epitope	Host species	Reactivity
FXP3/197	N-terminus	Mouse	Human, monkey, mouse
SPM579	N-terminus	Mouse	Human, monkey, mouse
3G3	N-terminus (mouse)	Mouse	Human, mouse
PCH101	Exon 1	Rat	Human, chimpanzee, rhesus, cynomolgus
1054C	Exon 1 (aa 1–71)	Rabbit	Human, mouse
16J4G6	Δ2	Mouse	Human
150D/E4	Exon 2	Mouse	Human, mouse, rat
FJK-16s	Exon 2	Rat	Human, mouse, rat, dog, pig, cow
376209	Exon 3–5 (aa 105–200)	Mouse	Human, mouse, rat
F-9	Exon 3–5 (aa 107–190)	Mouse	Human, mouse
206D	Exon 3–6 (aa 105–235)	Mouse	Human, baboon, rhesus, pigtailed macaque, cynomolgus
259D(/C7)	Exon 3–6 (aa 105–235)	Mouse	Human, chimpanzee, cynomolgus, rhesus, baboon
eBio7979	Exon 3–6	Mouse	Human, mouse
236A/E7	Exon 3–6	Mouse	Human, rhesus macaque, sooty mangabey, cynomolgus
D6O8C	Exon 8 (aa 293 ± x)	Rabbit	Human
D25D4	Exon 8 (aa 295 ± x)	Rabbit	Human
4F12F1	Exon 8–11 (aa 297–431)	Mouse	Human
22510	Exon 11 (aa 400–431)	Mouse	Human, mouse, rat
450	Exon 11 (aa 400–431)	Mouse	Human
nBcdbn 33622	Exon 11 (aa 400–431)	Mouse	Human
nBcdbn 561	Exon 11 (aa 400–431)	Mouse	Human
SP97	C-terminus	Rabbit	Human
TQ08	C-terminus	Rabbit	Human
SB151b	Full length	Mouse	Human
2A11G9,2A11C2	Unknown	Mouse	Human, mouse, rat
260E/F5	Unknown	Mouse	Human, mouse, rat
347B/F8	Unknown	Mouse	Human, non-human primate, rhesus
3B22D3	Unknown	Mouse	Human, mouse
3B22H0	Unknown	Mouse	Human, mouse
4C7	Unknown	Mouse	Human
5D8	Unknown	Mouse	Human
5H10L18	Unknown	Rabbit	Human, mouse
6H3C5H3	Unknown (mouse)	Mouse	Human, mouse, rat
8080	Unknown	Mouse	Human, mouse
99D04	Unknown	Mouse	Human, mouse
QDI202	Unknown	Rat	Human, chimpanzee, rhesus, cynomolgus
LS-C66372	Unknown	Mouse	Human, mouse

While alternative splicing of FOXP3 appears to add another layer of complexity to Treg cell biology, it can be of potential therapeutic value. First, the availability of whole transcriptome sequencing data will reveal associations of *FOXP3* splice variants with clinical diseases allowing faster diagnosis. Second, genetic disorders with impaired alternative splicing have previously been treated with ASOs in clinical trials (121). Along this line, ASOs that interfere with *FOXP3* pre-mRNA splicing may become a potent tool to affect cellular functions, as alternative splicing of *FOXP3* regulates development, proliferation, lineage stability and suppressive capacity of FOXP3+ cells. Thus, manipulation of alternative splicing to generate therapeutic FOXP3 isoform profiles could allow novel treatments to modulate Treg cell suppression, to control Th17 cell differentiation as well as to inhibit carcinoma proliferation in the future.

## Author Contributions

The author confirms being the sole contributor of this work and approved it for publication.

## Conflict of Interest Statement

The author has filed a patent application on FOXP3 isoform modification and declares no conflict of interest.
